# Circular RNA-circPan3 attenuates cardiac hypertrophy via miR-320-3p/HSP20 axis

**DOI:** 10.1186/s11658-023-00520-2

**Published:** 2024-01-03

**Authors:** Xinyu Fang, Xiang Ao, Dandan Xiao, Yu Wang, Yi Jia, Peiyan Wang, Mengyang Li, Jianxun Wang

**Affiliations:** School of Basic Medicine, Qingdao University, Qingdao, 266071 China

**Keywords:** Cardiac hypertrophy, m^6^A modification, Circular RNA, circPan3, miR-320-3p, HSP20

## Abstract

**Background:**

Circular RNAs are enriched in cardiac tissue and play important roles in the pathogenesis of heart diseases. In this study, we aimed to investigate the regulatory mechanism of a conserved heart-enriched circRNA, circPan3, in cardiac hypertrophy.

**Methods:**

Cardiac hypertrophy was induced by isoproterenol. The progression of cardiomyocyte hypertrophy was assessed by sarcomere organization staining, cell surface area measurement, and expression levels of cardiac hypertrophy markers. RNA interactions were detected by RNA pull-down assays, and methylated RNA immunoprecipitation was used to detect m^6^A level.

**Results:**

The expression of circPan3 was downregulated in an isoproterenol-induced cardiac hypertrophy model. Forced expression of circPan3 attenuated cardiomyocyte hypertrophy, while inhibition of circPan3 aggravated cardiomyocyte hypertrophy. Mechanistically, circPan3 was an endogenous sponge of miR-320-3p without affecting miR-320-3p levels. It elevated the expression of HSP20 by endogenously interacting with miR-320-3p. In addition, circPan3 was N6-methylated. Stimulation by isoproterenol downregulated the m^6^A eraser ALKBH5, resulting in N6-methylation and destabilization of circPan3.

**Conclusions:**

Our research is the first to report that circPan3 has an antihypertrophic effect in cardiomyocytes and revealed a novel circPan3-modulated signalling pathway involved in cardiac hypertrophy. CircPan3 inhibits cardiac hypertrophy by targeting the miR-320-3p/HSP20 axis and is regulated by ALKBH5-mediated N6-methylation. This pathway could provide potential therapeutic targets for cardiac hypertrophy.

**Graphical Abstract:**

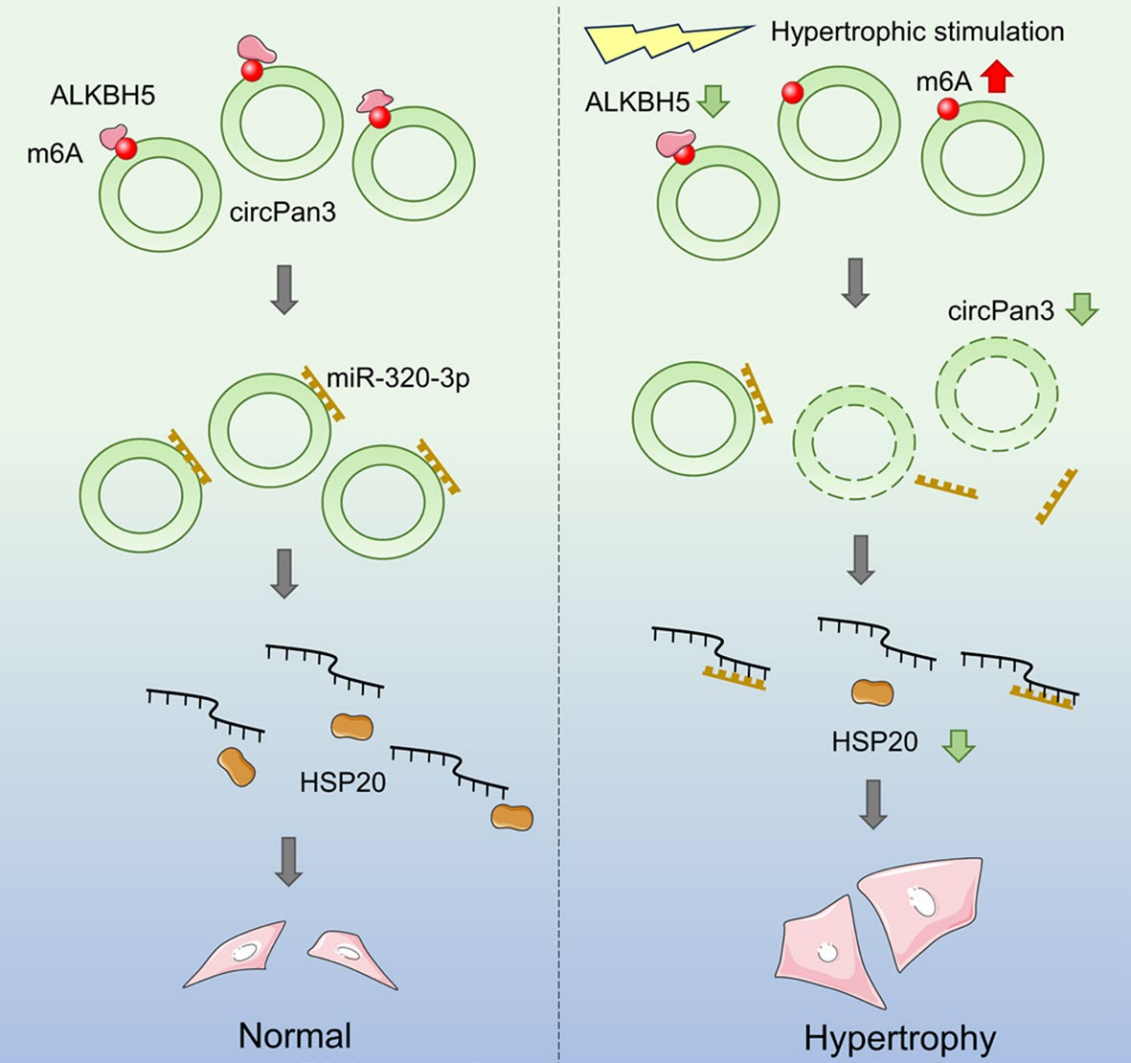

## Background

Pathological myocardial hypertrophy is the basic pathogenesis of various heart diseases. The progression of pathological hypertrophy could lead to heart failure and even sudden death. On the molecular biology level, pathological cardiac hypertrophy has been demonstrated to be related to abnormal expression of some cardiac hypertrophy-related genes and activation of signalling pathways including calcineurin, mTOR signalling, serine/threonine kinase calcium/calmodulin-dependent protein kinase type II (CaMKII), phosphoinositide 3-kinase (PI3K), alpha serine/threonine-protein kinase (Akt) and mitogen-activated protein kinase (MAPK) [1–4]. However, the molecular mechanism of cardiac hypertrophy has not been fully elucidated.

Noncoding RNAs including miRNAs and lncRNAs, have been reported to be involved in cardiac hypertrophy [5–8]. Circular RNAs (circRNAs) are new types of noncoding RNAs and generated by back-splicing of precursor mRNAs. They show extreme stability and post-transcriptionally regulate gene expression via multiple mechanisms [9, 10]. By means of high-throughput RNA sequencing, a large number of heart-enriched circRNAs have been identified [11–13]. Gradually, it has been revealed that dysregulation of these cardiac circRNAs is closely correlated with the progression of heart diseases, including ischaemic myocardial injury and cardiac fibrosis [14–17]. Remarkably, some members have shown significant regulatory effects in cardiac hypertrophy. For instance, a heart related circRNA (HRCR) controls cardiomyocyte hypertrophy by inhibiting miR-223 [18]. Circ-SIRT1 inhibits cardiac hypertrophy by promoting autophagy by activating SIRT1 [19]. CircRNA_000203 exacerbates cardiac hypertrophy by inhibiting miR-26b-5p and miR-140-3p [20]. CircRNA wwp1 plays a role in inhibiting cardiac hypertrophy by downregulating atrial natriuretic peptide (ANP) and miR-23a [21]. These findings suggest the regulatory potential of cardiac circRNAs in cardiac hypertrophy, which cannot be ignored.

Here, we report a cardiac hypertrophy related circRNA, which is generated from the 2nd-4th exons of the *Pan3* gene and named circPan3. The RNA-seq data showed that circPan3 is quite conserved and abundant in human and murine hearts [11], implying its fundamental role. Our group initially verified the existence of circPan3 in cardiomyocytes and demonstrated that circPan3 can protect cardiomyocytes from doxorubicin (DOX)-induced cardiotoxicity in previous study [22]. The present research focused on the role of circPan3 in cardiac hypertrophy. The results showed that circPan3 is downregulated in ISO-induced cardiomyocyte hypertrophy, it can inhibit cardiomyocyte hypertrophy by regulating the miR-320-3p/HSP20 axis. We further found that the expression of circPan3 in cardiac hypertrophy is affected by Alkylated DNA repair protein alkB homolog 5 (ALKBH5) mediated N6-methylation. This pathway could provide potential therapeutic targets for cardiac hypertrophy.

## Methods

### Cell culture, treatment and cell surface area measurement

Neonatal rat cardiomyocytes were isolated from 1 to 2-day-old Wistar rats and prepared as previously described [18]. Isoproterenol was used at a dose of 10 μM to induce cardiomyocyte hypertrophy [18]. The cell surface area measurement was performed as previously described [18]. Briefly, cardiomyocytes were fixed in 4% formaldehyde, then treated with 0.5% Triton X-100 (T8200, Solarbio, China) for 5 min. Then, the cells were incubated with 50 μg/ml fluorescent phalloidin-TRITC conjugate (CA1610, Solarbio, China) for 30 min at room temperature and visualized by a laser confocal microscopy (Zeiss LSM 510 META). The cell surface area was measured by ImageJ software. A total of 3 independent experiments were performed. The cell surface area of  ≥ 50 cardiomyocytes was detected in each experiment.

### Animal experiments

Eight-week-old adult male C57BL/6 mice with similar body weights were chosen for experiments. All experiments were carried out in accordance with the guidelines for Animal Experimentation of Qingdao University and approved by the Ethics Committee of Medical College of Qingdao University. Cardiac hypertrophy disease models were induced in the mice by chronic infusion of isoproterenol (ISO, Sigma, USA) for 14 days at a dose of 45 mg/kg/day with dorsally implanted minipumps (Alzet Model 1002, Alza Corp, USA). Normal control mice received the same volume of saline only. Adeno-associated virus (AAV) of circPan3 or control AAV were synthesized by HANBIO (China), and administered by direct injection to the caudal vein before ISO infusion (2 × 10^12^ moi). At the end of the treatment period, the pumps were surgically removed, and the mice were lightly anaesthetized and subjected to body weight measurement and echocardiographic assessment. Finally, the animals were sacrificed by injection of excessive anesthetics (200 mg/kg sodium pentobarbital). The hearts were isolated for weighing, detection of RNA and protein expression, and histological analysis.

### Histological analysis

Histological analysis of the hearts was carried out as previously described [18]. Briefly, hearts were excised, fixed in 10% formalin, embedded in paraffin, sectioned into 7 μm slices, and stained with hematoxylin–eosin (H&E, Spark Jade, China). To measure the cross-sectional area of the cardiomyocytes, the sections were stained with FITC-conjugated wheat germ agglutinin (WGA, Maokang Biotechnology, China).

### Echocardiographic assessment

Echocardiographic assessment was carried out on lightly anaesthetized mice by using a VINNO 6 LAB high-resolution system (VINNO, China) equipped with a 23 MHz × 10^−23^ L scan head. Systolic left ventricular internal diameter (LVIDs), diastolic left ventricular internal diameter (LVIDd) and left ventricular ejection fraction (LVEF) were measured. Fractional shortening (FS) of the left ventricular diameter was calculated as (LVIDd − LVIDs)/LVIDd × 100%.

### RNA pull-down assay

An RNA pull-down assay was performed to detect the interaction between circPan3 and miR-320-3p, as previously described [22].

Briefly, for the forward RNA pull-down assay, a 5ʹ-biotin-labelled circPan3 probe, which specifically targets the junction region of circPan3, as well as scrambled control 5ʹ-biotin-labelled probes, were synthesized and purchased from Sangon Biotech (China). The probes were incubated with streptavidin agarose beads for 2 h at 4 °C to generate probe-coated beads. Cells were washed with PBS and then incubated with lysis buffer (20 mM Tris–HCl, 200 mM NaCl, 2.5 mM MgCl_2_, 0.05% IgepalCA630, I8896-50 ml, Sigma, USA, 60 U/mL RNase inhibitor, RM21401, ABclonal, China, 1 mM DTT, protease inhibitor, Solarbio, China, pH 7.5) on ice for 30 min. The lysates of primary cardiomyocytes were precleared by centrifugation. The supernatant was pooled and incubated with streptavidin agarose beads at 4 °C for 3 h. The beads were washed once with the ice-cold lysis buffer, three times with low-salt buffer (20 mM Tris–HCl, 150 mM NaCl, 2 mM EDTA, 0.1% SDS, 1% Triton X-100, pH 8.0) and once with high salt buffer (20 mM Tris–HCl, 500 mM NaCl, 2 mM EDTA, 0.1% SDS, 1% Triton X-100, T8200, Solarbio, China, pH 8.0). All the buffers mentioned above were prepared with RNase-free water. The probe-coupled RNA was eluted with TRIzol. The levels of miR-320-3p and circPan3 were analyzed using qPCR. The sequence of the circPan3 probe was 5ʹ-bio-ACCTCCATCCATTCCGGGAACTTCCTTCTCTGG-3ʹ. The scrambled control probe was 5ʹ-bio-AAATGGCTTCGCAACCGAAT-3ʹ.

For the reverse RNA pull-down assay, we synthesized miR-320-3p mimics and scrambled single-strand RNAs, which were labelled with 5ʹ-biotin. Cardiomyocytes were transfected with biotinylated miRNA mimics and harvested 24 h after transfection. The cells were washed with PBS followed by brief vortexing, and incubated in lysis buffer on ice for 30 min. The lysates were precleared by centrifugation, and 50 μL of the samples was aliquoted for input. The remaining lysates were incubated with streptavidin agarose beads for assay as described above. The sequence of the biotin-labelled miR-320-3p mimic was 5ʹ-bio-AAAAGCUGGGUUGAGAGGGCGA-3ʹ. The sequence of the biotin-labelled scrambled control RNA oligo was 5ʹ-bio-GAAGGGUAGGACCAAAGUGGUG-3ʹ.

### Vector construction and transfection

The circPan3 vector was synthesized as previously described, with slight modifications [23]. The circPan3 sequence along with the ALU elements utilized in circRNA Mini Vector (Addgene plasmid # 60648) were inserted into the pcDNA3.1 (+) plasmid. Transfection of plasmids was performed using Lipofectamine 3000 (Thermo Fisher, Waltham, MA, USA) according to the manufacturer’s instructions.

### RNA interference (RNAi)

Small interfering RNA (siRNA) oligonucleotides specific for circPan3, HSP20 and ALKBH5 were designed using Ambion’s siRNA design tool, and purchased from GenePharma Co. Ltd (Shanghai, China). Transfection of siRNAs was performed using Lipofectamine 3000 (Thermo Fisher, Waltham, MA, USA) according to the manufacturer’s instructions. The specificity of the oligonucleotides was confirmed through comparison with nucleotide collections in GenBank using nucleotide BLAST. The detailed sequences of the siRNAs were as follows: circPan3 siRNA: 5ʹ-AGAAGGAAGUUCCCGGAAUTT-3ʹ, negative control: 5ʹ-UUCUCCGAACGUGUCACGUTT-3ʹ, ALKBH5 siRNA: 5ʹ-GCCUCAGGACAUCAAAGAATT-3ʹ, negative control: 5ʹ-UUCUCCGAACGUGUCACGUTT-3ʹ, HSP20 siRNA: 5ʹ-CUGGAUGUGAAGCACUUCUTT-3ʹ, negative control: 5ʹ-UUCUCCGAACGUGUCACGUTT-3ʹ.

### Cell transfection with miRNA mimics or inhibitors

MiR-320-3p mimics and antisense oligonucleotides (inhibitors) used to inhibit endogenous miR-320-3p expression were synthesized by GenePharma Co. Ltd. Cells were transfected with miRNA mimics (100 nM) or inhibitors (100 nM) using Lipofectamine 3000 (Thermo Fisher) according to the manufacturer’s instructions. The detailed sequences of the miRNA mimics and inhibitors were as follows: miR-320-3p mimic: 5ʹ-AAAAGCUGGGUUGAGAGGGGCGA-3ʹ, control mimic: 5ʹ-UUCUCCGAACGUGUCACGUTT-3ʹ. Antisense oligonucleotides (inhibitors) were used to inhibit the expression of endogenous miR-320-3p. The inhibitor sequence was 5ʹ-UCGCCCUCUCAACCCAGCUUU-3ʹ. The control sequence was 5ʹ-CAGUACUUUUGUGUAGUACAA-3ʹ.

### Western blotting

Cardiomyocytes were incubated with lysis buffer (150 mM NaCl, 1% Triton X-100, 1% sodium deoxycholate, 0.1% SDS and protease inhibitors, Solarbio, China) on ice for 30 min. Protein was separated by SDS-PAGE. Electrophoresis was run for 20 min under 80 V followed by 90 min under 120 V. Subsequently, the protein was transferred from gel to PVDF membrane. The membrane was blocked by 5% nonfat milk dissolved in TBST for 1 h at room temperature. The blocked membrane was incubated with primary antibodies (anti-HSP20, ab188864, Abcam, 1:1000, anti-GAPDH, Boster, BM1623, 1:5000, anti-ALKBH, 16837-1-AP, Proteintech, 1:1000, anti-METTL3, 15073-1-AP, proteintech,1:1000, anti-METTL14, 26158-1-AP, Proteintech, 1:1000, anti-ALKBH5, 16837-1-AP, Proteintech, 1:1000, anti-FTO, 27226-1-AP, Proteintech,1:1000, anti-WTAP, 10200-1-AP, Proteintech, 1:1000) at 4 °C overnight. The membrane was washed 3 times with TBST and incubated with secondary antibody dissolved in 5% nonfat milk for 1 h. Western blots were visualized using the ECL kit (P10100A, NCM Biotech, China).

### Divergent PCR

The head-to-tail junction part of circPan3 was validated by PCR with divergent primers. Convergent primers were used as controls. The specificity of the PCR amplification was confirmed by agarose gel electrophoresis. The detailed sequences of the primers are shown in Table 1.

**Table 1:** The sequences of primers used in this study

Assay	Primer name/target	Species	Sequence (5ʹ–3ʹ)
Divergent PCR	CircPan3-forward	Rat	TAGAAACAAAGTATCCCTTGATGCA
Divergent PCR	CircPan3-reverse	Rat	TTAAAGCACCTCCATCCATTCC
Convergent PCR	CircPan3-forward	Rat	CGGAATGGATGGAGGTGCTT
Convergent PCR	CircPan3-reverse	Rat	ATCAAGGGATACTTTGTTTCTACCG
qPCR	CircPan3-forward	Rat	TAAATGACAGTGCCAAGCCATAC
qPCR	CircPan3-reverse	Rat	GCATCAAGGGATACTTTGTTTCTAC
qPCR	Pan3-forward	Rat	TTCAGCACCAGCTTCATTGGAGTGA
qPCR	Pan3-reverse	Rat	CCTGTGTATGGCTTGGCACTGTCA
qPCR	METTL3-forward	Rat	GGAAGCACGCTGCCTCAGATGTT
qPCR	METTL3-reverse	Rat	GGACTGTTCCTTGGCTGTTGTGGTA
qPCR	METTL14-forward	Rat	TCTTCGGGAGGGACAGCACTATCAG
qPCR	METTL14-reverse	Rat	ACGGTCAGACTTGGATTTGGGAGGA
qPCR	FTO-forward	Rat	GGCTGTGGAAGAAGATGGAGAGTGT
qPCR	FTO-reverse	Rat	GCTGTGCTGGTAGAGTTCGGACAA
qPCR	WTAP-forward	Rat	CAGTACCAGCAGCAGCAGTCTCAAG
qPCR	WTAP-reverse	Rat	CCTCCCTGTGAAATCCAGACCCAGA
qPCR	ALKBH5-forward	Rat	ACGCAGTGACTACGAGGAGCATCA
qPCR	ALKBH5-reverse	Rat	TGGAGCACTCATCCTGGCTGAAGA
qPCR	GAPDH-forward	Rat	GCCCATCACCATCTTCCAGGAG
qPCR	GAPDH-reverse	Rat	GAAGGGGCGGAGATGATGAC
qPCR	MiR-320-3p-forward	Rat	ACACTCCAGCTGGGAAAAGCTGGGTTGAGA
qPCR	MiR-320-3p-reverse	Rat	TGGTGTCGTGGAGTCG
qPCR	U6-forward	Rat	CTCGCTTCGGCAGCACA
qPCR	U6-reverse	Rat	AACGCTTCACGAATTTGCGT
qPCR	ANP-forward	Rat	AGGAGAAGATGCCGGTAGAAGA
qPCR	ANP-reverse	Rat	CCGAGAGCACCTCCATCTCT
qPCR	BNP-forward	Rat	TGCTCCTGCTTTTCCTTAATCTG
qPCR	BNP-reverse	Rat	TTGAGAGCTGTCTCTGAGCCATT
qPCR	β-MHC- forward	Rat	AGCCTCCAGAGTTTGCTGAA
qPCR	β-MHC- reverse	Rat	TTGATGAGGCTGGGGGTTCTG
qPCR	CircPan3-forward	Mouse	GATCTTCTGACTTCATCTGCTTCATC
qPCR	CircPan3-reverse	Mouse	TACTTTGTTTCTACAGGGCTTCCAA
qPCR	GAPDH- forward	Mouse	CAGTGGCAAAGTGGAGATTGTTG
qPCR	GAPDH- reverse	Mouse	TCGCTCCTGGAAGATGGTGAT
qPCR	MiR-320-3p-forward	Mouse	CCAAAAGCTGGGTTGAGAGG
qPCR	MiR-320-3p-reverse	Mouse	CCAGTGCAGGGTCCGAGGT
qPCR	U6-forward	Mouse	CGCTTCGGCAGCACATATACTA
qPCR	U6-reverse	Mouse	GGAACGCTTCACGAATTTGC
qPCR	ANP-forward	Mouse	AGGCCATATTGGAGCAAATCC
qPCR	ANP-reverse	Mouse	GCTTCCTCAGTCTGCTCACTCA
qPCR	BNP-forward	Mouse	CTGTCCCAGATGATTCTGTTTCTG
qPCR	BNP-reverse	Mouse	GGCCATTTCCTCCGACTTTT
qPCR	β-MHC- forward	Mouse	GGAGGCTCTGATCTCTCAGCTAA
qPCR	β-MHC- reverse	Mouse	GTTCCCTCAGCAGGTCACAATC
RT	MiR-320-3p-RT	Rat	CTCAACTGGTGTCGTGGAGTCGGCAATTCAGTTGAGTCGCCCTC
RT	MiR-320-3p-RT	Mouse	GTCGTATCCAGTGCAGGGTCCGAGGTATTCGCACTGGATACGACTCGCCC

### Quantitative real-time PCR (qPCR)

Total RNA was extracted with TRIzol (AC0101-B, Spakzol Reagent, China) and reverse-transcribed with a HiScript III RT SuperMix for qPCR (+ gDNA WIper) reverse transcription kit (Vazyme, R323-01, China). The gene expression level was analyzed by quantitative real-time PCR (qPCR). The expression of miR-320-3p was normalized to that of U6. The levels of circPan3 and other mRNAs were normalized to that of GAPDH. The detailed sequences of the primers are shown in Table 1.

### RNase R digestion

Five milligrams of total RNA was incubated with or without RNase R (3 U μg^−1^, Epicentre Biotechnologies) for 15 min at 37 ℃. RNA was subsequently purified by TRIzol reagent. The levels of CircPan3 and Pan3 mRNA were analyzed by qPCR.

### Methylated RNA immunoprecipitation (MeRIP)

MeRIP was performed according to a previous method [24] with modifications. Recombinant protein A/G plus agarose beads (Thermo Scientific, 20423, USA) were washed with immunoprecipitation (IP) buffer (10 mM Tris–HCl, 150 mM NaCl and 0.1% (vol/vol) Igepal CA-630) and then blocked with 0.5 mg/mL BSA at 4 °C for 60 min. The m^6^A-specific antibody (AB208577, Abcam, USA) and beads were incubated in IP buffer at 4 °C for 1 h. Mouse IgG was selected as a control. The beads were washed three times with IP buffer to remove the uncoated antibodies. The antibody-coated beads were then incubated with 8 μg of total RNA from rat cardiomyocytes, which was dissolved in IP buffer, at 4 °C for 2 h. N^6^-modified RNAs were purified by TRIzol. The m^6^A enrichment of circPan3 was assessed by qPCR.

### Statistical analysis

Data are expressed as the mean ± S.E.M. of at least three independent experiments. Statistical analysis for comparison of two groups was performed using two-tailed unpaired Student’s *t*-test. For comparison of more than two groups, one-way analysis of variance (ANOVA) followed by Tukey post hoc test was performed. Values of *P* < 0.05 and *P* < 0.01 were considered significant and extremely significant, respectively.

## Results

### CircPan3 is correlated with ISO-induced cardiomyocyte hypertrophy

**Fig. 1:**
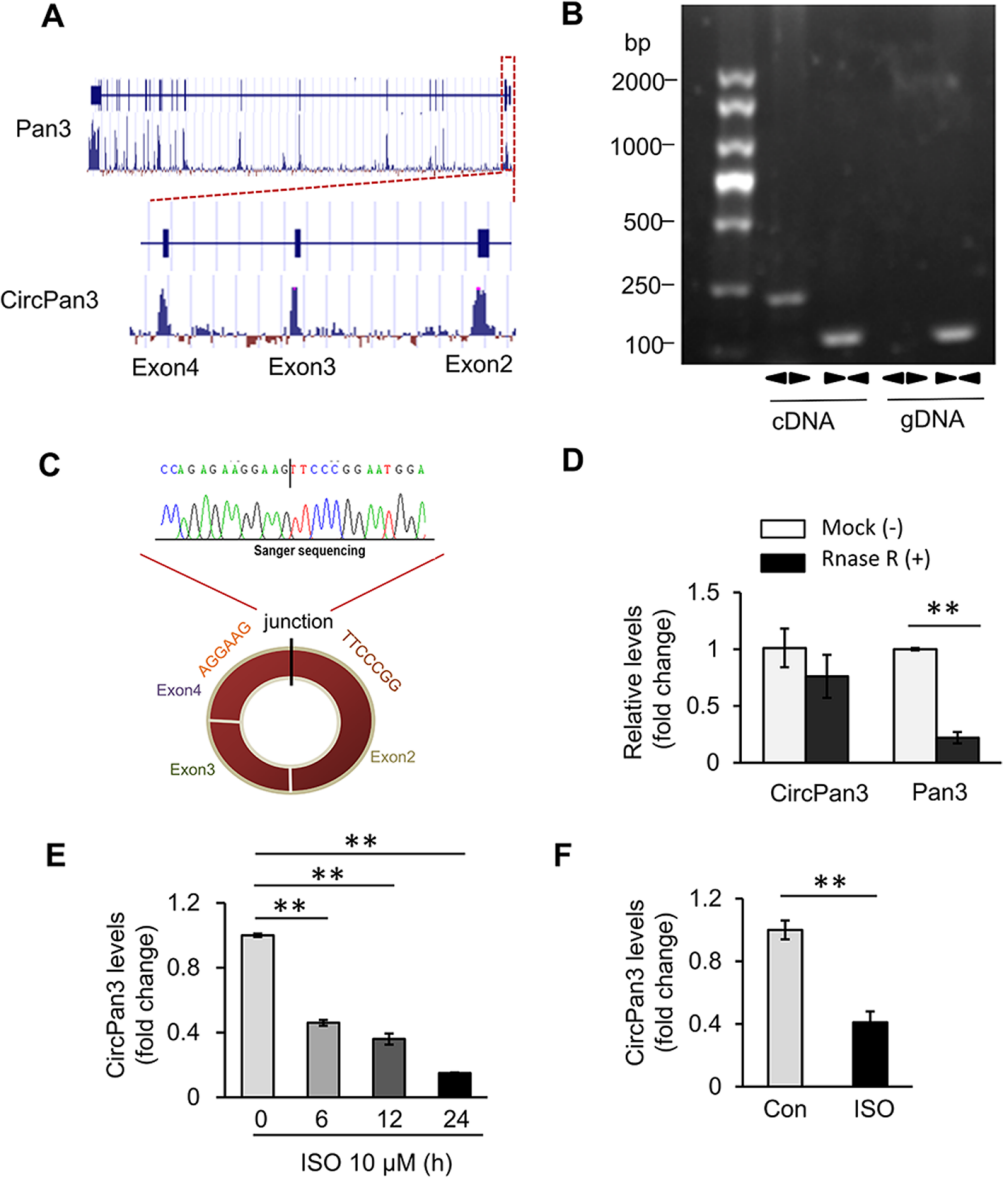
CircPan3 is a conserved circRNA in the rodent cardiac tissue. **A** CircPan3 was generated from the 2nd–4th exons of the *pan3* gene. Sequence analysis of PhyloP showed that circPan3 is conserved. **B** CircPan3 was amplified from the cDNA of rat cardiomyocytes by divergent primers, while it could not be amplified from gDNA. **C** Sanger-Seq validated the head-to-tail junction of the circPan3 sequence. **D** CircPan3 is resistant to digestion by RNase R. RNAs from rat cardiomyocytes were incubated with RNase R or buffer only (Mock). After digestion, the RNAs were purified. The levels of circPan3 and Pan3 mRNA were analyzed by qPCR. **P < 0.01 versus Mock. *n* = 3. **E** The expression of circPan3 in cardiomyocytes decreased upon treatment with ISO. Cardiomyocytes were treated with 10 μM ISO. The level of circPan3 was analyzed by qRT-PCR. **P < 0.01 versus 0 h. *n* = 3. **F** CircPan3 is downregulated in the murine cardiac tissues of cardiac hypertrophy. A mouse cardiac hypertrophy model was induced by treatment with ISO for 2 weeks. The level of circPan3 in mouse hearts was analyzed by qPCR. **P < 0.01. *n* = 6

According to RNA-Seq data [11–13], circPan3 originates from exons 2–4 of the *pan3* gene, which is relatively conserved (Fig. 1A). In our previous study, circPan3 was found to have a protective effect on DOX-induced cardiotoxicity [22]. We wondered whether circPan3 was also involved in cardiac hypertrophy. We performed divergent PCR to verify the circular structure of circPan3. A clear single band was amplified from cDNA by divergent primers (Fig. 1B). The sanger sequence showed that the sequence of the amplification product corresponded to the sequencing data and contained the junction site of back-splicing (Fig. 1C). It was further found that circPan3 was resistant to degradation after treatment with RNase R (Fig. 1D). To test whether circPan3 was correlated with cardiac hypertrophy, ISO was used to induce a cardiac hypertrophy model in vitro and in vivo. The expression level of circPan3 in cardiomyocytes upon treatment with ISO showed a downwards trend (Fig. 1E). Similarly, circPan3 was also downregulated in ISO-induced cardiac hypertrophy in vivo (Fig. 1F). These results suggest that circPan3 is a cardiac circular RNA that may be involved in cardiac hypertrophy.

### CircPan3 inhibits ISO-induced cardiomyocyte hypertrophy

**Fig. 2:**
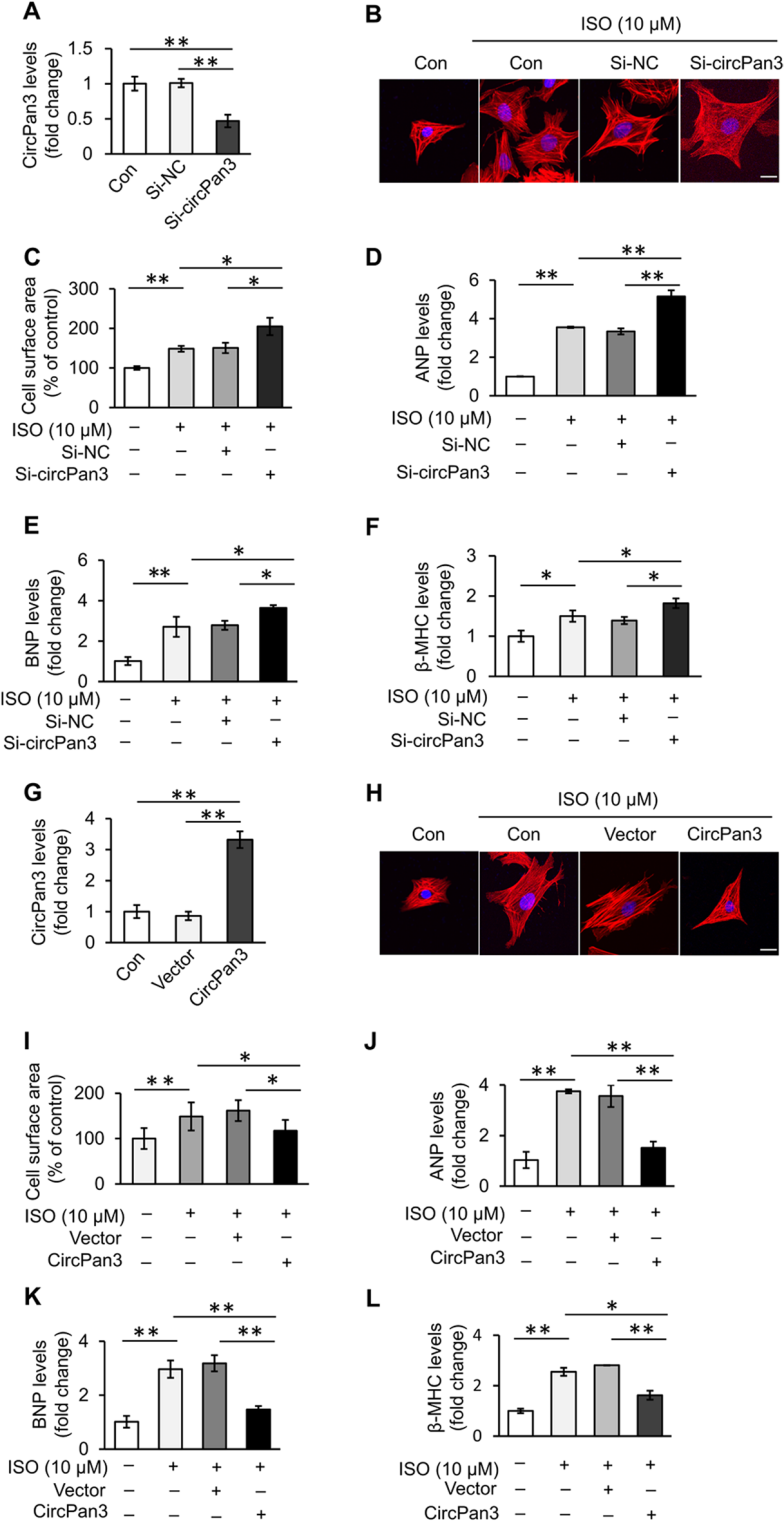
CircPan3 inhibits ISO-induced cardiomyocyte hypertrophy. **A** The expression level of circPan3 in cardiomyocytes transfected with circPan3-siRNA (si-circPan3) or its scramble control (si-NC) was analyzed by qPCR. *P < 0.01. *n* = 3. **B**–**F** Knockdown of circPan3 aggravates ISO-induced cardiomyocyte hypertrophy. Cardiomyocytes were transfected with si-circPan3 or si-NC. Forty-eight hours after ISO treatment, cardiomyocyte hypertrophy was assessed by **B** sarcomere organization (bar = 20 μm), **C** cell surface area measurement and level of **D** ANP, **E** BNP and **F** β-MHC. The expression level of ANP, BNP and β-MHC was analyzed by qPCR. *P < 0.05, **P < 0.01. *n* = 3. **G** The expression level of circPan3 in cardiomyocytes transfected with the circPan3 expression vector or empty vector was analyzed by qPCR. **P < 0.01. *n* = 3. **H–L** Forced expression of circPan3 inhibits ISO-induced cardiomyocyte hypertrophy. Cardiomyocytes were transfected with the circPan3 expression vector or empty vector. Forty-eight hours after ISO treatment, cardiomyocyte hypertrophy was assessed by **H** sarcomere organization (bar = 20 μm), **I** cell surface area measurement and level of **J** ANP, **K** BNP and **L** β-MHC. The expression level of ANP, BNP and β-MHC was analyzed by qPCR. *P < 0.05, **P < 0.01. *n* = 3

Next, to explore the regulatory roles of circPan3 in cardiomyocyte hypertrophy, loss-of-function experiments were applied. First, siRNAs specifically targeting the junction site of circPan3 (si-circPan3) were designed and synthesized to silence endogenous circPan3. The knockdown efficiency of si-circPan3 was verified (Fig. 2A). Phalloidin staining showed that the cell surface area of cardiomyocytes transfected with si-circPan3 was significantly larger than that of cardiomyocytes in the control group upon ISO treatment (Fig. 2B, C). Correspondingly, the expression of the hypertrophic indicators including ANP, brain natriuretic peptide (BNP) and beta myosin heavy chain (β-MHC) in cardiomyocytes was also increased by knockdown of circPan3 (Fig. 2D–F). On the other hand, transfection with the circPan3 expression vector markedly increased the level of circPan3 in cardiomyocytes (Fig. 2G). Overexpression of circPan3 significantly abolished ISO-induced cardiomyocyte hypertrophy (Fig. 2H–L). The above experimental data indicate that circPan3 can inhibit cardiomyocyte hypertrophy in vitro.

### CircPan3 interacts with miR-320-3p

**Fig. 3:**
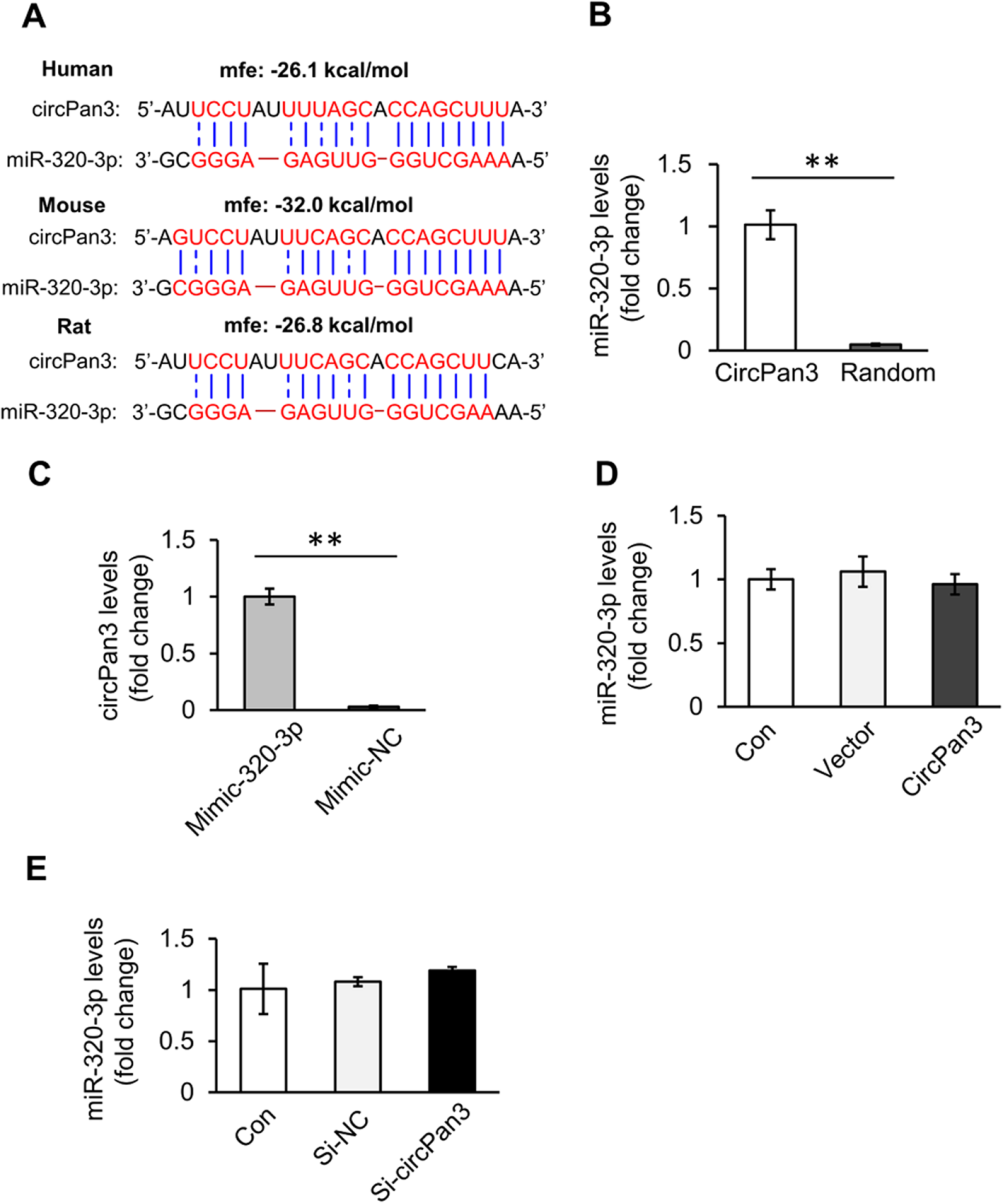
CircPan3 interacts with miR-320-3p. **A** A conserved miR-320-3p binding site exists in the circPan3 sequence. Minimum free energy (mfe) was calculated by RNAhybrid. **B** MiR-320-3p is captured by circPan3. The levels of captured miR-320-3p and U6 by the biotin-labelled circPan3 probe or scrambled control probe (Random) were analyzed by qPCR. The relative pellet/input ratios were calculated. **P < 0.01. *n* = 3. **C** CircPan3 is captured by miR-320-3p. The levels of captured circPan3 and GAPDH by biotin-labelled miR-320-3p mimics or negative control mimics (Mimic-NC) were analyzed by qPCR. The relative pellet/input ratios were calculated. **P < 0.01. *n* = 3. **D** Overexpression of circPan3 does not affect miR-320-3p levels in cardiomyocytes. Cardiomyocytes were transfected with the circPan3 expression vector or empty vector. The expression level of miR-320-3p was analyzed by qPCR. *n* = 3. **E** Knockdown of circPan3 does not affect miR-320-3p levels in cardiomyocytes. Cardiomyocytes were transfected with siRNAs. The expression level of miR-320-3p was analyzed by qPCR. *n* = 3

The miRNA sponge is one of the major roles of circRNA. According to the data in circBank [25] and ENCORI [26], miR-320-3p is a potential target of circPan3. RNAhybrid prediction suggested that a potential conserved miR-320-3p binding site with high affinity was present in the circPan3 sequence (Fig. 3A). To verify the interaction between miR-320-3p and circPan3, RNA pull-down assays were performed. Compared with the control probe, the biotin-labelled circPan3 probe-captured RNA fraction contained much more miR-320-3p (Fig. 3B). Moreover, reversed pull-down showed that biotin-labelled miR-320-3p mimics could capture more circPan3 than the negative control mimics (Fig. 3C). Further studies showed that neither overexpression nor knockdown of circPan3 affected miR-320-3p levels (Fig. 3D, E). Taken together, these results show that circPan3 acts as a sponge for miR-320-3p without regulating its expression.

### MiR-320-3p regulates cardiomyocyte hypertrophy

**Fig. 4:**
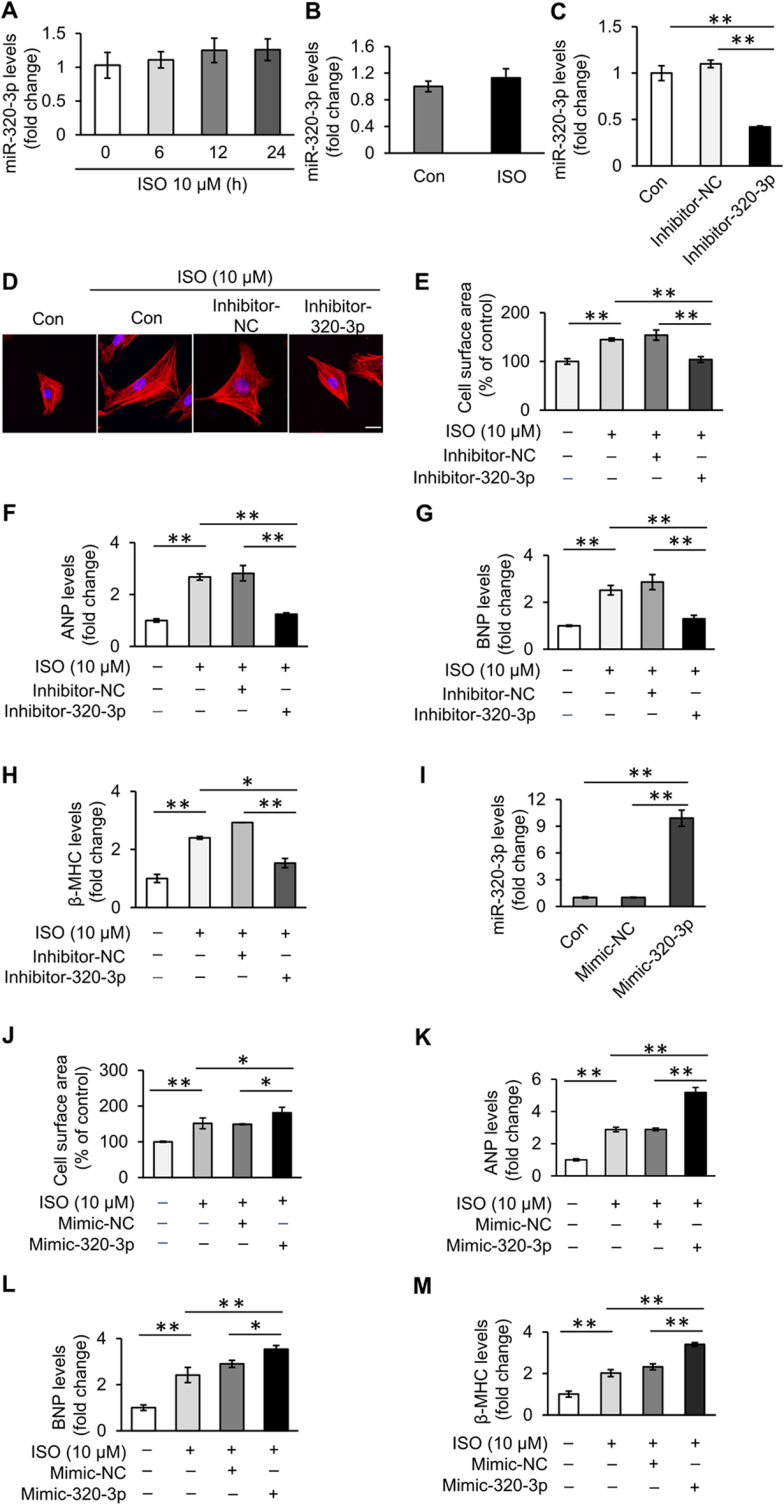
MiR-320-3p promotes ISO-induced cardiomyocyte hypertrophy. **A**, **B** No change in miR-320-3p levels in cardiac hypertrophy. **A** Cardiomyocytes were treated with 10 μΜ ISO. The expression level of miR-320-3p was analyzed by qPCR. *n* = 3. **B** The expression level of miR-320-3p in rat hypertrophic cardiac tissue was analyzed by qPCR. *n* = 3. **C** The expression level of miR-320-3p in cardiomyocytes transfected with miR-320-3p inhibitor or negative control inhibitor (inhibitor-NC) was analyzed by qPCR. **P < 0.01. *n* = 3. **D-H** Knockdown of miR-320-3p inhibits cardiomyocyte hypertrophy. Cardiomyocytes were transfected with miR-320-3p inhibitor or negative control inhibitor. Forty-eight hours after ISO treatment, cardiomyocyte hypertrophy was assessed by **D** sarcomere organization (bar = 20 μm), **E** cell surface area measurement and level of **F** ANP, **G** BNP and **H** β-MHC. The expression level of ANP, BNP and β-MHC was analyzed by qPCR. *P < 0.05, **P < 0.01. *n* = 3. **I** The expression level of miR-320-3p in cardiomyocytes transfected with miR-320-3p mimic or negative control mimic (mimic-NC) was analyzed by qPCR. **P < 0.01. *n* = 3. **J–M** MiR-320-3p aggravates ISO-induced cardiomyocyte hypertrophy. Cardiomyocytes were transfected with miR-320-3p mimic or control mimic. Forty-eight hours after ISO treatment, cardiomyocyte hypertrophy was assessed by **J** cell surface area measurement and level of **K** ANP, **L** BNP and **M** β-MHC. The expression level of ANP, BNP and β-MHC was analyzed by qPCR. *P < 0.05, **P < 0.01. *n* = 3

Next, we tested the function of miR-320-3p in cardiomyocyte hypertrophy. Although the expression of miR-320-3p was not significantly affected by ISO in vitro or in vivo (Fig. 4A, B), knockdown of miR-320-3p decreased the cell surface area of cardiomyocytes and the expression level of the hypertrophic indicators in cardiomyocytes upon ISO treatment (Fig. 4C–H). Conversely, transfection of miR-320-3p mimics in cardiomyocytes increased the expression level of the hypertrophic indicators and the cell surface area (Fig. 4I–M). These results indicate that miR-320-3p promotes cardiomyocyte hypertrophy.

### MiR-320-3p aggravates cardiomyocyte hypertrophy by targeting HSP20

**Fig. 5:**
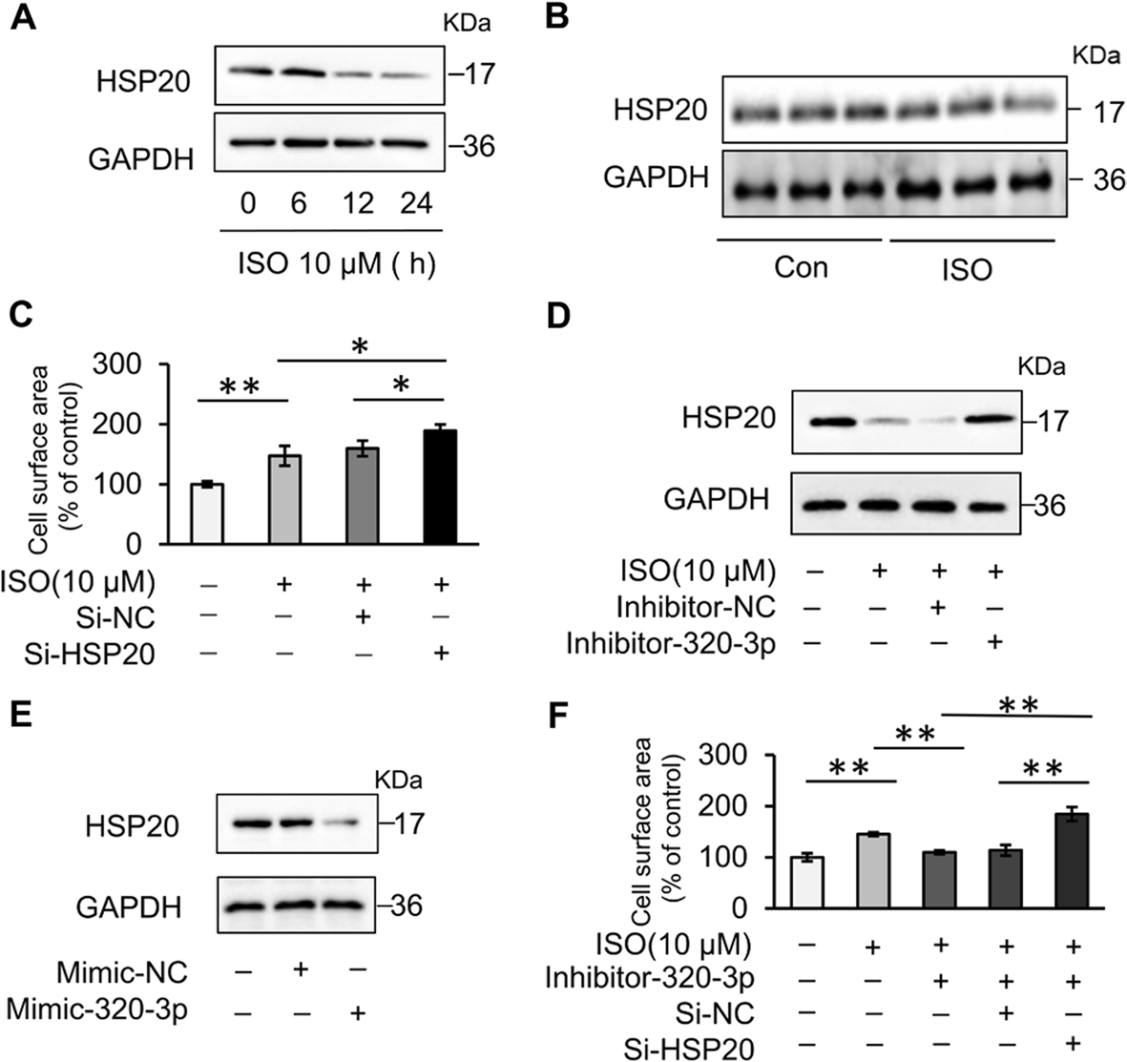
MiR-320-3p acts by targeting HSP20. **A-B** HSP20 is downregulated in cardiac hypertrophy. **A** The expression of HSP20 in cardiomyocytes treated with 10 μM ISO was analyzed by immunoblotting. *n* = 3. **B** The expression of HSP20 in the myocardial tissue of the ISO-induced mouse cardiac hypertrophy model was analyzed by immunoblotting. *n* = 6. **C** Silencing of HSP20 aggravates ISO-induced cardiomyocyte hypertrophy. Cardiomyocytes were transfected with HSP20 siRNA or its scramble control (si-NC) and then exposed to 10 μM ISO for 48 h. Cardiomyocyte hypertrophy was assessed by cell surface area measurement, *P < 0.05, **P < 0.01. *n* = 3. **D–E** The expression of HSP20 in cardiomyocytes was decreased by miR-320-3p. **D** Cardiomyocytes were transfected with miR-320-3p inhibitor or negative control inhibitor (Inhibitor-NC) and then treated with 10 μM ISO for 24 h. **E** Cardiomyocytes were transfected with miR-320-3p mimic or control mimic. The expression of HSP20 was analyzed by immunoblotting. **F** The antihypertrophic effect of the miR-320-3p inhibitor is abolished by HSP20-siRNA. Cardiomyocytes were co-transfected with miR-320-3p inhibitor and HSP20-siRNA and then exposed to 10 μM ISO for 48 h. Cardiomyocyte hypertrophy was assessed by cell surface area measurement. **P < 0.01. *n* = 3

According to previous studies, HSP20 might be a critical downstream target of miR-320-3p [27]. To test whether miR-320-3p also regulates cardiomyocyte hypertrophy through HSP20, the following studies were carried out. First, we observed that the expression of HSP20 in cardiomyocytes was decreased upon ISO treatment (Fig. 5A). Correspondingly, HSP20 was also downregulated in ISO-induced hypertrophic myocardial tissue from mice (Fig. 5B), suggesting that HSP20 might be involved in cardiac hypertrophy. Loss-of-function experiments demonstrated the protective effect of HSP20 in cardiomyocyte hypertrophy (Fig. 5C). More importantly, the protein level of HSP20 was increased after inhibition of miR-320-3p (Fig. 5D), but decreased after overexpression of miR-320-3p (Fig. 5E). Simultaneous knockdown of HSP20 significantly reversed the phenomenon caused by the miR-320-3p inhibitor (Fig. 5F). Therefore, miR-320-3p is highly likely to promote the occurrence of cardiomyocyte hypertrophy by inhibiting HSP20, which is consistent with our hypothesis.

### CircPan3 protects cardiomyocytes from hypertrophy by mediating the miR-320-3p/HSP20 pathway

**Fig. 6:**
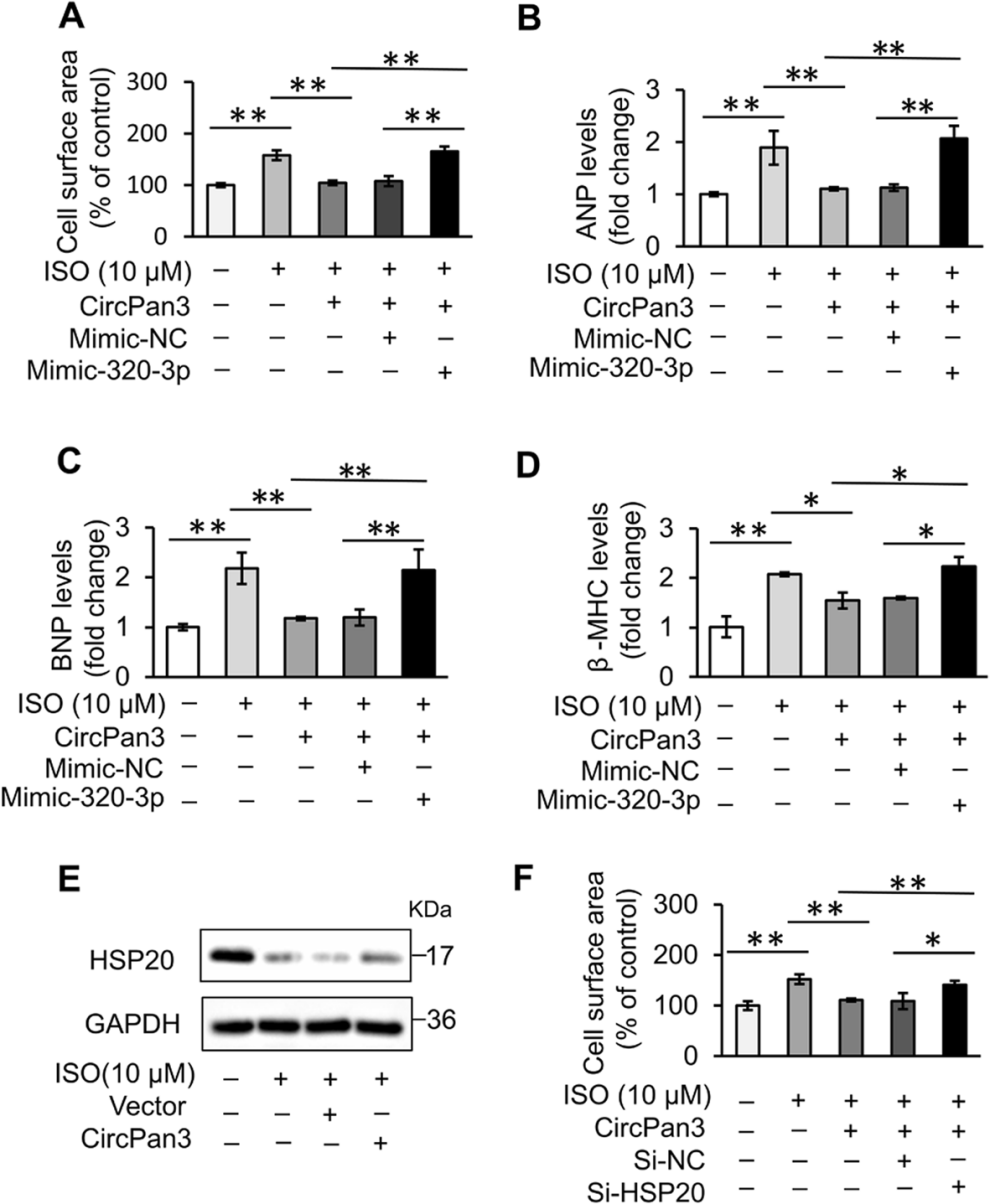
CircPan3 inhibits ISO-induced cardiac hypertrophy by regulating the miR-320-3p/HSP20 axis. **A–D** CircPan3 inhibits cardiomyocyte hypertrophy by regulating miR-320-3p. Cardiomyocytes were co-transfected with the circPan3 expression vector and miR-320-3p mimic (or control mimics). Cells were treated with 10 μM ISO for 48 h. Cardiomyocyte hypertrophy was assessed by **A** cell surface area measurement and level of **B** ANP, **C** BNP and **D** β-MHC. The expression level of ANP, BNP and β-MHC was analyzed by qPCR. *P < 0.05, **P < 0.01. *n* = 3. **E** CircPan3 regulates the expression of HSP20. Cardiomyocytes were transfected with the circPan3 expression vector or empty vector and then treated with 10 μM ISO for 24 h. The expression of HSP20 was analyzed by immunoblotting. **F** Knockdown of HSP20 counteracts the effect of circPan3 in cardiomyocyte hypertrophy. Cardiomyocytes were co-transfected with the circPan3 expression vector and HSP20-siRNA (or its scramble control) and then exposed to 10 μM ISO for 48 h. Cardiomyocyte hypertrophy was assessed by cell surface area measurement. *P < 0.05, **P < 0.01. *n* = 3

Based on the above results, we hypothesized that circPan3 might function in cardiomyocyte hypertrophy by regulating the miR-320-3p/HSP20 pathway. To verify this hypothesis, cardiomyocytes were first co-transfected with the circPan3 expression vector and miR-320-3p mimics. It could be observed that circPan3 inhibited cardiomyocyte hypertrophy, while miR-320-3p eliminated the effect of circPan3 (Fig. 6A–D). We further verified that HSP20 was regulated by circPan3. Compared with the control group, overexpression of circPan3 increased the protein level of HSP20 in cells (Fig. 6E). Correspondingly, knockdown of HSP20 in cardiomyocytes abolished the effect of circPan3 overexpression, which reverted the cell surface area to hypertrophic conditions (Fig. 6F). Taken together, these data suggest that circPan3 protects cardiomyocytes from hypertrophy by inhibiting the miR-320-3p/HSP20 pathway.

### ALKBH5-modulated m^6^A modification affects the stability of circPan3

**Fig. 7:**
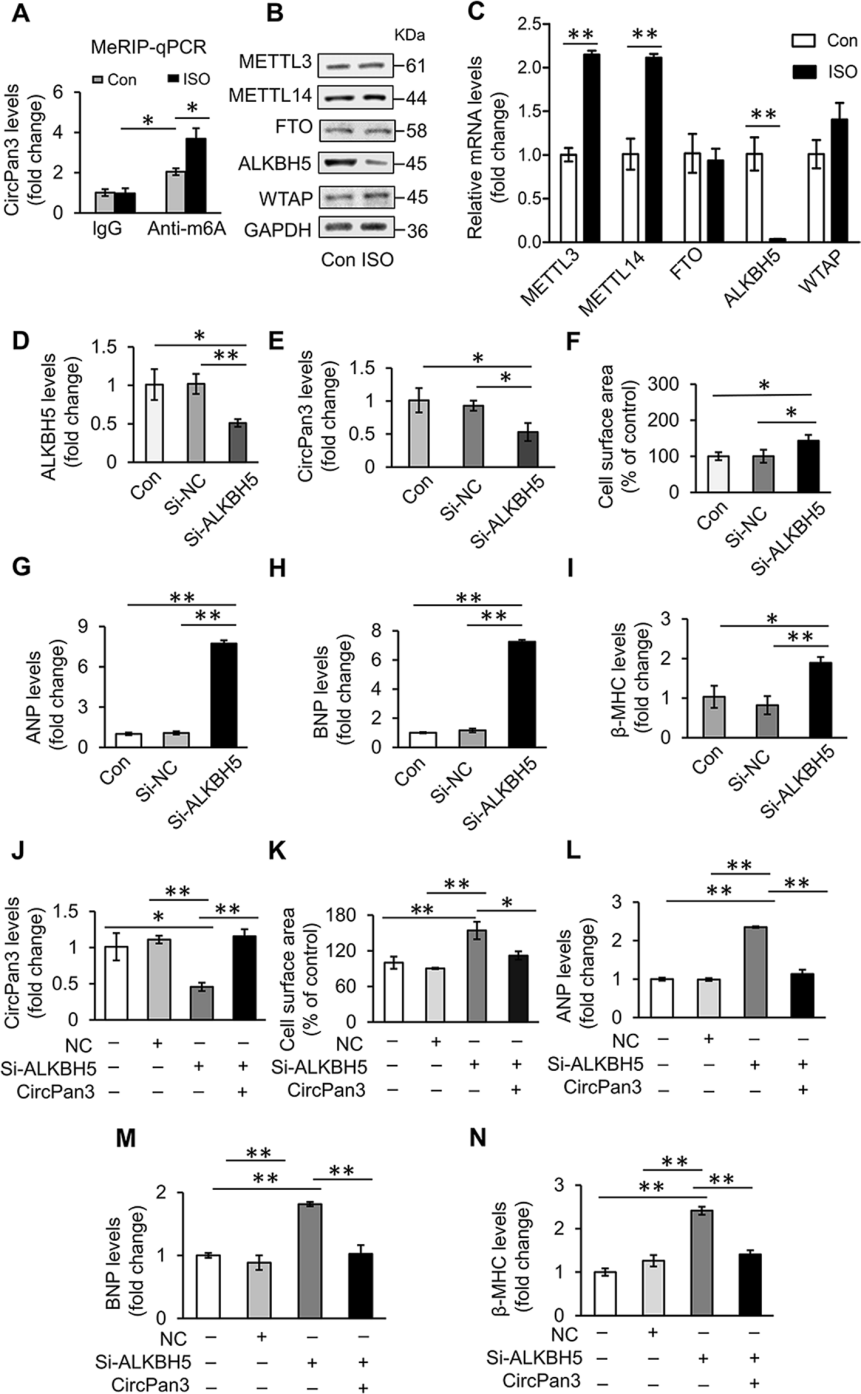
ALKBH5 regulates the stability of circPan3 through m^6^A modification. **A** The level of N6-methylated circPan3 in cardiomyocytes is increased by ISO-stimulation. The lysate of cardiomyocytes treated with ISO or saline (con) was incubated with m^6^A antibody or control IgG-coated agarose beads. The N6-methylated RNAs captured by antibodies were purified by TRIzol. The circPan3 level was analyzed by qPCR. *P < 0.05. *n* = 3. **B, C** ALKBH5 is significantly downregulated in cardiomyocyte hypertrophy. Cardiomyocytes were treated with 10 μΜ ISO for 24 h. The protein and mRNA levels of m^6^A writers and erasers in cardiomyocytes were analyzed by **B** immunoblotting and **C** qPCR, respectively. **P < 0.01. *n* = 3. **D–E** Knockdown of ALKBH5 reduces the expression of circPan3 in cardiomyocytes. Cardiomyocytes were transfected with ALKBH5-siRNA for 24 h. The expression of ALKBH5 **D** and circPan3 **E** was measured by qPCR. *P < 0.05, **P < 0.01. *n* = 3. **F–I** Silencing of ALKBH5 promotes cardiomyocyte hypertrophy. Cardiomyocyte hypertrophy was assessed by **F** cell surface area measurement and level of **G** ANP, **H** BNP and **I** β-MHC. The expression level of ANP, BNP and β-MHC was analyzed by qPCR. *P < 0.05, **P < 0.01. *n* = 3. **J–N** Overexpression of circPan3 abolishes cardiomyocyte hypertrophy induced by knockdown of ALKBH5. **J** Cardiomyocytes were co-transfected with ALKBH5-siRNA and the circPan3 vector for 24 h. The expression of circPan3 was measured by qPCR. *P < 0.05, **P < 0.01. *n* = 3. Cardiomyocyte hypertrophy was assessed by **K** cell surface area measurement and level of **L** ANP, **M** BNP and **N** β-MHC. The expression level of ANP, BNP and β-MHC was analyzed by qPCR. *P < 0.05, **P < 0.01. *n* = 3

Next, we examined the reason why circPan3 is dysregulated in cardiomyocyte hypertrophy. M^6^A is a type of internal chemical modification that has been reported to affect the biogenesis, processing and stability of circRNAs [28–31] Moreover, m^6^A was found to be closely related to cardiac hypertrophy [32]. Coincidently, several potential m^6^A sites in circPan3 were predicted by SRAMP [33]. Therefore, we hypothesized that the downregulation of circPan3 in cardiomyocyte hypertrophy was caused by m^6^A modification. The MeRIP assay was used to test the N^6^-methylation level of circPan3. The results showed that circPan3 is enriched in m^6^A antibody-captured RNA fractions. The m^6^A modification level of circPan3 was further increased in ISO-treated cardiomyocytes (Fig. 7A). Considering that N^6^-methylation is regulated by a series of writer and eraser proteins, we wondered whether some members of these m^6^A regulators affect the N^6^-methylation level of circPan3. To explore whether the hypermethylation of circPan3 in cardiomyocyte hypertrophy was caused by upregulation of m^6^A writers or downregulation of m^6^A erasers, we first detected the expression of central m^6^A writers, including Methyltransferase-like protein 3 (METTL3), Methyltransferase-like protein 14 (METTL14) and WT1-associated protein (WTAP), as well as central m^6^A erasers including Fat mass and obesity-associated protein (FTO) and ALKBH5 [34], in cardiomyocytes upon ISO treatment. It was found that ALKBH5 was the only m^6^A eraser that was significantly downregulated at both the mRNA and protein levels (Fig. 7B, C). Therefore, we first utilized ALKBH5 siRNA to interfere with ALKBH5 in cardiomyocytes (Fig. 7D) and tested whether there is a regulatory role of ALKBH5 in circPan3 and cardiomyocyte hypertrophy. It was found that knockdown of ALKBH5 decreased the expression level of circPan3 (Fig. 7E). ALKBH5 has been reported to regulate cardiomyocyte proliferation and cardiac regeneration [35]. We speculated that ALKBH5 also plays a role in cardiomyocyte hypertrophy. It was shown that knockdown of ALKBH5 in cardiomyocytes significantly increases the cell surface area and the levels of the hypertrophic indicators (Fig. 7F–I). Simultaneous overexpression of circPan3 abolished cardiomyocyte hypertrophy caused by knockdown of AKLBH5 (Fig. 7J–N). From these results, it can be suggested that ALKBH5 regulates the stability of circPan3 through N^6^-methylation to affect the process of cardiomyocyte hypertrophy.

### CircPan3 alleviates cardiac hypertrophy in vivo

**Fig. 8:**
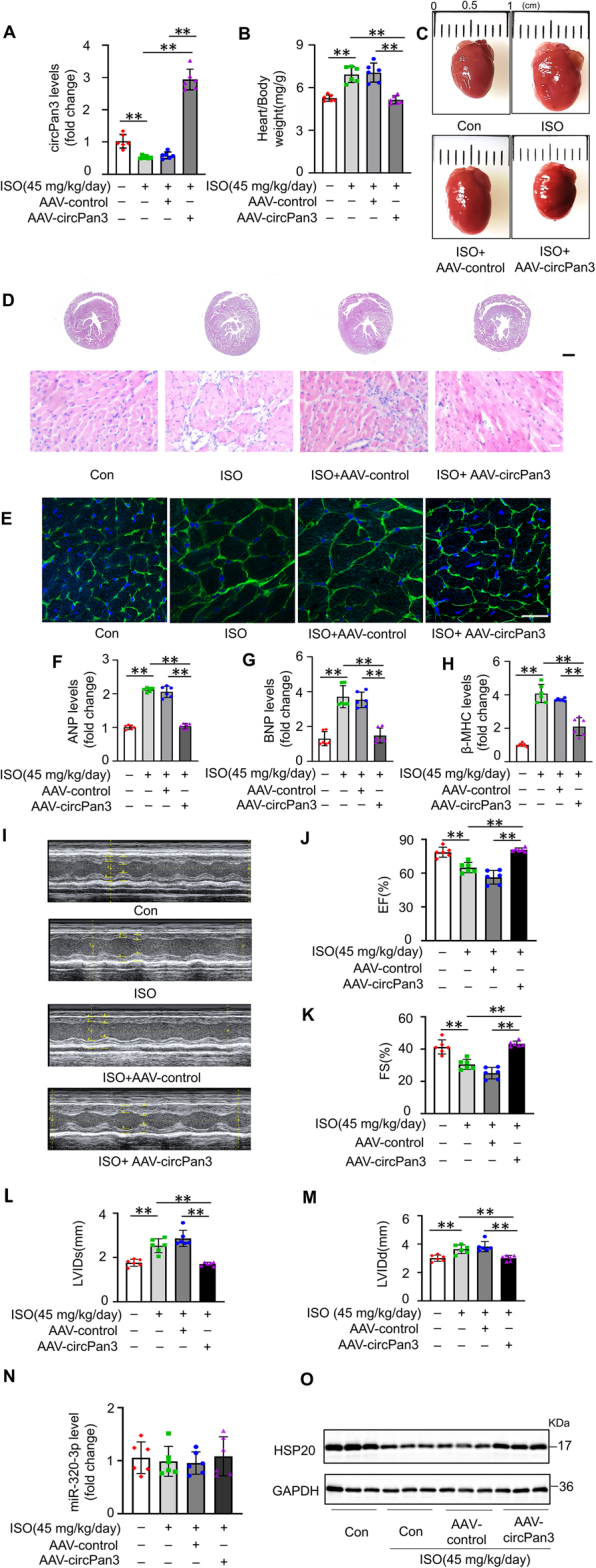
CircPan3 inhibits cardiac hypertrophy in vivo*.* **A** CircPan3 AAV rescues ISO-induced downregulation of circPan3 in cardiac hypertrophy. The level of circPan3 in mouse hearts was analyzed by qPCR. **P < 0.01, *n* = 6. **B–H** CircPan3 alleviates cardiac hypertrophy. **B **Heart weight/body weight ratio of mice. **P < 0.01, *n* = 6. **C** Representative images of mouse whole hearts. **D** H&E staining of the heart sections (bar = 20 μm). **E** WGA staining of the heart sections (bar = 25 μm). **F–H** The expression level of ANP, BNP and β-MHC in mouse hearts was analyzed by qPCR. *P < 0.01. *n* = 6. **I–M** CircPan3 rescues impaired heart function. **I** Echocardiography of the mouse hearts. **J** EF, **K** FS, **L** LVIDs and **M** LVIDd were detected. *P < 0.01. *n* = 6. **N** The expression level of miR-320-3p in mouse hearts was analyzed by qPCR. *n* = 6. **O** The expression level of HSP20 in mouse hypertrophic cardiac tissue was analyzed by immunoblotting

To verify the effect of circPan3 in vivo, mice were infected with circPan3 AAV and then subjected to chronic infusion of ISO to establish a cardiac hypertrophy model. After 2 weeks of ISO infusion, the expression of circPan3 in mouse hearts was significantly reduced, resembling the phenomena in vitro, while circPan3 AAV significantly infected the heart and effectively reversed ISO-induced downregulation of circPan3 (Fig. 8A). Next, the degree of cardiac hypertrophy was evaluated. Compared with saline-infused mice, ISO-infused mice showed obviously higher heart weights (Fig. 8B) and heart sizes (Fig. 8C). HE and WGA staining indicated an increase in left ventricle wall thickness (Fig. 8D) and cardiomyocyte surface area (Fig. 8E) in ISO-infused mice respectively. The expression of the hypertrophic indicators in the heart was also remarkably induced by ISO (Fig. 8F–H). Echocardiographic assessment further showed that ISO infusion reduced heart function (Fig. 8I–M). These results indicated that the cardiac hypertrophy model was successfully established. However, simultaneous infection with circPan3 AAV significantly repressed the increase in heart weight (Fig. 8B), heart size (Fig. 8C), left ventricle thickness (Fig. 8D), cardiomyocyte surface area (Fig. 8E) and the hypertrophic indicators expression (Fig. 8F–H). Moreover, circPan3 AAV prevented the impairment of heart function (Fig. 8I–M). The results verified that circPan3 inhibits cardiac hypertrophy in vivo. In addition, the expression of miR-320-3p and HSP20 was also detected. It can be observed that the level of miR-320-3p was not significantly changed under ISO stimulation, while overexpression of circPan3 also did not affect the expression of miR-320-3p (Fig. 8N). On the other hand, HSP20 was obviously downregulated by ISO. CircPan3 rescued the reduction in HSP20 (Fig. 8O). Therefore, it can be concluded that the effects of ISO and circPan3 on the expression patterns of miR-320-3p and HSP20 in vivo are similar to those in vitro, further suggesting the significance of the circPan3/miR-320-3p/HSP20 axis in cardiac hypertrophy.

## Discussion

In this study, we demonstrated that circPan3 inhibits cardiac hypertrophy by targeting the miR-320-3p/HSP20 axis. Moreover, we revealed the underlying mechanism of circPan3 dysregulation in cardiomyocyte hypertrophy: CircPan3 is N6-methylated. The decrease in the m^6^A eraser ALKBH5 in cardiomyocyte hypertrophy causes elevated m^6^A levels and destabilization of circPan3.

Other circRNAs annotated as “circPan3” have also been reported to regulate the self-renewal of intestinal stem cells, drug resistance in acute myeloid leukemia, and myocardial ischemia/reperfusion injury, respectively [36–38]. For instance, Zhang et al. demonstrated that circPan3 suppresses cardiomyocyte autophagy and apoptosis by targeting miR-421/Pink1 [38]. Although these circRNAs are also transcribed from the *Pan3* gene, their sequences are distinct from the one reported by us, suggesting that they are functionally independent. Our studies showed that circPan3 generated from exons 2–4 can inhibit DOX-induced cardiotoxicity [22] and ISO-induced cardiomyocyte hypertrophy, indicating its unique protective effect.

MiR-320-3p has been reported to function in various diseases by regulating cell proliferation, death and differentiation [39–46]. Importantly, miR-320-3p is enriched in heart tissues and was revealed to aggravate ischemic myocardial injury and cardiomyocyte death [27, 47]. However, it was unclear whether miR-320-3p also functioned in cardiac hypertrophy. Our data showed that miR-320-3p can aggravate ISO-induced cardiomyocyte hypertrophy while silencing of endogenous miR-320-3p protects cardiomyocytes from hypertrophy, indicating the prohypertrophic effect of miR-320-3p.

The miRNA sponge is one of the major roles of circRNAs [48, 49]. A fraction of circRNAs with multiple miRNA binding sites show remarkably strong inhibition of target miRNAs. For instance, ciRS-7 contains as many as 60 conserved miR-7 target sites [49]. Nevertheless, one miRNA binding site with relatively high affinity is sufficient for circRNAs to function as miRNA sponges [18, 50, 51]. Our analysis discovered a high-affinity miR-320-3p binding site in the circPan3 sequence, which is relatively conserved across species, implying that there might be indispensable regulation of miR-320-3p by circPan3 being conserved during evolution. Generally, the regulation of miRNAs by circRNAs can be divided into two types: miRNA can either be degraded, or repressed without affecting their level. Once the targeted miRNAs are perfectly complementary to the sequence of circRNAs, they are released from the RNA-induced silencing complex (RISC) and degraded by AGO2-associated nucleases, such as the regulation of CDR1as on miR-671 [52]. Other miRNAs showing lower sequence complementarity with targeted circRNAs are usually stable in RISC and will not recruit the RNA decay machinery. Therefore, the interaction of circRNAs only neutralizes the repression of miRNAs on downstream targets [53]. Our results showed that circPan3 can increase the expression of miR-320-3p-targeted HSP20 without affecting miR-320-3p levels, indicating that circPan3 acts as an adsorbent of miR-320-3p and does not cause miR-320-3p degradation. This is possibly due to the incomplete complementation between circPan3 and miR-320-3p. On the other hand, circPan3 might also function via other mechanisms in addition to binding miR-320-3p. One circRNA can act as a sponge for multiple miRNAs. For instance, circNCX1 (also named circSLC8a1) is the most abundant circRNA in the heart and is highly associated with AGO2, implying its high affinity for miRNAs. CircNCX1 has been demonstrated to mainly function by targeting miR-133 [14], while it has also been shown to interact with other miRNAs, including miR-16, miR-208-5p, let-7, miR-34a, miR-1, miR-103 and miR-30b [54]. In addition, some circRNAs simultaneously act as protein scaffolds and miRNA sponges. For example, circNfix reinforced the interaction of Ybx1 (Y-box binding protein 1) with Nedd4l (neural precursor cell-expressed developmentally down-regulated 4-like), as well as binding miR-214 to inhibit cardiomyocyte proliferation [55]. It remains to be further detected whether circPan3 regulates cardiomyocyte hypertrophy by interacting with other miRNAs or proteins.

The biogenesis of circRNA is modulated by RNA binding proteins (RBPs) involved in pre-mRNA splicing [9, 10]. Quaking (QKI) is a splicing factor that shows a protective effect in ischemic myocardial injury and DOX-induced cardiotoxicity [56–59]. Noticeably, QKI also regulates circRNA formation [60]. QKI has been reported to be involved in DOX-induced cardiotoxicity by generating Ttn-derived circRNAs [57]. Our previous study has demonstrated that the production of circPan3 is regulated by QKI. Downregulation of QKI in cardiomyocytes by DOX decreases the expression of circPan3. However, the expression of QKI in cardiac hypertrophy or heart failure was not shown to be significantly changed according to published omics analysis [61–65]. This result suggested that the dysregulation of circPan3 in cardiac hypertrophy might be caused by a QKI-independent mechanism.

M^6^A is the methylation at the N^6^ position of adenine and the most abundant eukaryotic RNA modification, it is involved in almost every process of post-translational regulation, including RNA splicing, export, translation and degradation, to affect gene expression [34, 66, 67]. An increasing number of studies have demonstrated that m^6^A modification regulates a variety of biological and pathological processes, including embryonic development, immune reactions, and cancer progression [68–70]. Moreover, some recent evidence has shown that m^6^A is also involved in the biogenesis, translocation and degradation of circRNAs [28–31]. Noticeably, m^6^A is closely correlated with cardiac hypertrophy [32, 35, 71]. The global m^6^A level of transcriptomes in cardiomyocytes was increased under cardiac hypertrophy. Elevation of m^6^A level by overexpression of the m^6^A writer METTL3 can cause cardiac hypertrophy [32], while m^6^A eraser FTO attenuates cardiac dysfunction in mice with pressure overload-induced heart failure via N6-demethylation [72]. These results indicate that dysregulation of m^6^A will lead to aberrant expression of cardiac hypertrophy-related genes. Bioinformatics prediction showed that there are m^6^A sites in circPan3. Thus, we wondered whether dysregulation of circPan3 during cardiomyocyte hypertrophy is caused by m^6^A. The MeRIP-qPCR results indicate that circPan3 in cardiomyocytes is N^6^-methylated. Moreover, the m^6^A level of circPan3 can be increased by ISO treatment. We further demonstrated a significant decrease in the m^6^A eraser ALKBH5 in cardiomyocyte hypertrophy, which is consistent with the elevated N6-methylation of circPan3. ALKBH5 has been reported to be involved in hypoxia/reoxygenation-induced cardiomyocyte autophagy and cardiomyocyte proliferation [35, 73]. However, its role in cardiac hypertrophy is unclear. Our results showed that knockdown of ALKBH5 causes downregulation of circPan3 and aggravation of cardiomyocyte hypertrophy, suggesting that ALKBH5 regulates cardiomyocyte hypertrophy via m^6^A-dependent destabilization of circPan3. M^6^A has been reported to mediate the cleavage of circRNAs. The m^6^A reader YTHDF2 can recognize the m^6^A site in circRNAs, recruit RNase P/MRP, which is bridged by heat-responsive protein 12 (HRSP12), and cleave the circRNAs [31]. Therefore, the level of circPan3 in cardiomyocyte hypertrophy is likely to be associated with the demethylase activity of ALKBH5, as well as YTHDF2-mediated m^6^A recognition and RNA degradation. However, further verification is needed.

HSP20 is one of ten members of the small heat shock protein family, which is upregulated under stress conditions and thought to play a key role in cell survival [74]. HSP20 has been shown to be essential for cells to combat oxidative stress and damage by regulating the activity of multiple protein kinases, cleaning denatured and aggregated proteins, promoting protein folding or prolonging the half-life of growth factors [75–79]. HSP20 has been reported to inhibit cardiac hypertrophy and myocardial remodeling [80–82] and is a highly conserved target of miR-320-3p [27]. Our results showed that the expression of HSP20 is decreased in cardiomyocyte hypertrophy and repressed by miR-320-3p, which is consistent with previous studies. We further confirmed that HSP20 is regulated by circPan3/miR-320 in cardiomyocyte hypertrophy, revealing a novel upstream regulatory mechanism of HSP20.

## Conclusions

This study revealed a novel regulatory pathway in cardiac hypertrophy, which consists of circPan3, miR-320-3p and HSP20. Our results revealed the protective effect of circPan3 in cardiomyocyte hypertrophy and its regulatory mechanism. We also demonstrated that the expression of circPan3 in cardiomyocyte hypertrophy is affected by ALKBH5-modulated m^6^A methylation. In summary, our research provides a new therapeutic target for cardiac hypertrophy and helps to further elucidate the pathogenesis of myocardial hypertrophy and heart failure.

## Data Availability

Data sharing is not applicable to this article as no datasets were generated or analysed during the current study.

## Abbreviations

CaMKII: Calcium/calmodulin-dependent protein kinase type II
PI3K: Phosphoinositide 3-kinase
Akt: Alpha serine/threonine-protein kinase
MAPK: Mitogen-activated protein kinase
CircRNAs: Circular RNAs
ANP: Atrial natriuretic peptide
BNP: Brain natriuretic peptide
β-MHC: Beta myosin heavy chain
DOX: Doxorubicin
ISO: Isoproterenol
AAV: Adeno-associated virus
H&E: Hematoxylin-eosin
WGA: Wheat germ agglutinin
LVIDs: Systolic left ventricular internal diameter
LVIDd: Diastolic left ventricular internal diameter
LVEF: Left ventricular eject fraction
RNAi: RNA interference
SiRNA: Small interfering RNA
qPCR: Quantitative real-time PCR
MeRIP: Methylated RNA Immunoprecipitation
IP: Immunoprecipitation
Si-circPan3: SiRNA specifically targeting the junction site of circPan3
RISC: RNA-induced silencing complex
Ybx1: Y-box binding protein 1
Nedd4l: Neural precursor cell-expressed developmentally down-regulated 4-like
RBPs: RNA binding proteins
QKI: Quaking
METTL3: Methyltransferase-like protein 3
FTO: Fat mass and obesity-associated protein
HRSP12: Heat-responsive protein 12
ALKBH5: Alkylated DNA repair protein alkB homolog 5
METTL14: Methyltransferase-like protein 14
WTAP: WT1-associated protein
